# Cardiac Transcription Factor Nkx2.5 Is Downregulated under Excessive *O*-GlcNAcylation Condition

**DOI:** 10.1371/journal.pone.0038053

**Published:** 2012-06-15

**Authors:** Hoe Suk Kim, Ji Soo Woo, Hyun Jung Joo, Woo Kyung Moon

**Affiliations:** 1 The Institute of Radiation Medicine, Medical Research Center, Seoul National University, Jongno-gu, Seoul, Korea; 2 Department of Radiology, Seoul National University Hospital, Jongno-gu, Seoul, Korea; 3 Department of Biomedical Science, College of Medicine, Seoul National University, Seoul, Jongno-gu, Seoul, Korea; University of Western Ontario, Canada

## Abstract

Post-translational modification of proteins with *O*-linked N-acetylglucosamine (*O*-GlcNAc) is linked the development of diabetic cardiomyopathy. We investigated whether Nkx2.5 protein, a cardiac transcription factor, is regulated by *O*-GlcNAc. Recombinant Nkx2.5 (myc-Nkx2.5) proteins were reduced by treatment with the *O*-GlcNAcase inhibitors STZ and *O*-(2-acetamido-2-deoxy-D-glucopyroanosylidene)-amino-*N*-phenylcarbamate; PUGNAC) as well as the overexpression of recombinant *O*-GlcNAc transferase (OGT-flag). Co-immunoprecipitation analysis revealed that myc-Nkx2.5 and OGT-flag proteins interacted and myc-Nkx2.5 proteins were modified by *O*-GlcNAc. In addition, Nkx2.5 proteins were reduced in the heart tissue of streptozotocin (STZ)-induced diabetic mice and *O*-GlcNAc modification of Nkx2.5 protein increased in diabetic heart tissue compared with non-diabetic heart. Thus, excessive *O*-GlcNAcylation causes downregulation of Nkx2.5, which may be an underlying contributing factor for the development of diabetic cardiomyopathy.

## Introduction

Cyclical post-translational modification of proteins with *O*-linked *N*-acetylglucosamine (*O*-GlcNAc) is tightly regulated by the juxtaposed actions of *O*-GlcNAc transferase (OGT) and *O*-GlcNAc-selective *N*-acetylglucosaminidase (*O*-GlcNAcase), and serves as a nutrient and stress sensor [1], [2]. There exists a reciprocal relation between *O*-GlcNAcylation and phosphorylation on protein serine or threonine residues in regulating protein functions and stability [3]. *O*-GlcNAcylation affects protein–protein interactions, protein activity and stability [4]–[7].

Glycosylation with *O*-GlcNAc contributes to the etiology of diverse diseases including diabetes. In diabetic patients, the development of cardiomyopathy is attributable to hyperglycemia and increased modification of proteins with *O*-GlcNAc [8]–[11]. The mechanisms linking *O*-GlcNAcylation to cardiomyopathy are not known well.

Nkx2.5 is a homeobox-containing cardiac transcription factor that is highly expressed by cardiomyocytes and has well-established roles in cardiac development and disease [12]–[16]. We previously reported that excessive *O*-GlcNAcylation impairs the differentiation of Nkx2.5/GFP-knock-in ES cells into cardiac cells [17], implying that Nkx2.5 proteins were regulated by *O*-GlcNAc during cardiogenesis. Little is know about the Nkx2.5 protein function and stability by post-translational modification. It is reported that phosphorylation of Nlx2.5 protein by casein kinase II increased activity through increased DNA binding [18]. Recently, Small ubiquitin-like modifiers (SUMO) conjugation stabilized the formation of Nkx2.5-containing complexes and enhanced transcriptional activity of Nkx2.5 proteins [19]. However, glycosylation of Nkx2.5 protein with *O*-GlcNAc has not been reported.

Here, we investigated whether Nkx2.5 proteins post-translationally were modified by *O*-GlcNAc and the influence of the *O*-GlcNAc modification on Nkx2.5 stability in cells treated with *O*-GlcNAcase inhibitors (STZ and *O*-(2-acetamido-2-deoxy-D-glucopyroanosylidene)-amino-*N*-phenylcarbamate; PUGNAC) or the overexpression of OGT as well as STZ-induced diabetic heat tissues.

## Results

### Nkx2.5 Protein Decreased in the Heart Tissue of STZ-induced Diabetic Mice

**Figure 1:**
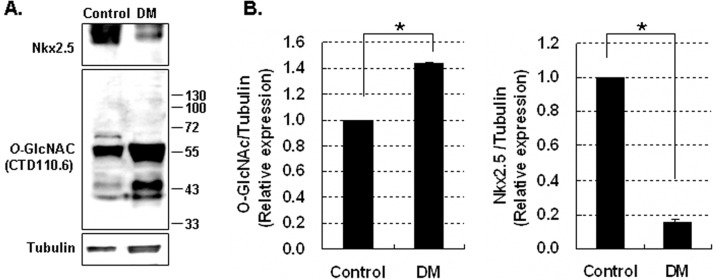
Expression of Nkx2.5 in the heart tissue of diabetic mice (DM). (A) Immunoblotting analysis of Nkx2.5 and *O*-GlycNAcylated proteins in the heart tissue of DM intraperitoneally injected with streptozotocine (180 mg/kg body weight). (B) Graphs showed relative *O*-GlcNAc (left) and Nkx2.5 (right) levels normalized to the tubulin levels in DM and control. Nkx2.5 protein in diabetic heart of mice was significantly decreased. *O*-GlcNAcylation and Nkx2.5 protein levels were compared in DM *vs* control. All values are presented as the mean±standard error of three independent experiments. *p<0.05 DM vs. control.

We investigated the levels of Nkx2.5 proteins in heart tissue isolated from diabetic mice and non-diabetic mice. Nkx2.5 protein levels were assessed by immunoblotting of homogenated diabetic heart tissue collected 5 weeks after treatment with STZ. In diabetic heart tissue, *O*-GlcNAcylation of proteins was higher while Nkx2.5 protein levels were lower than in non-diabetic heart tissue (Fig. 1A and B).

### Nkx2.5 Protein was Decreased by Treatment with O-GlcNacase Inhibitor STZ or PUGNAc

**Figure 2:**
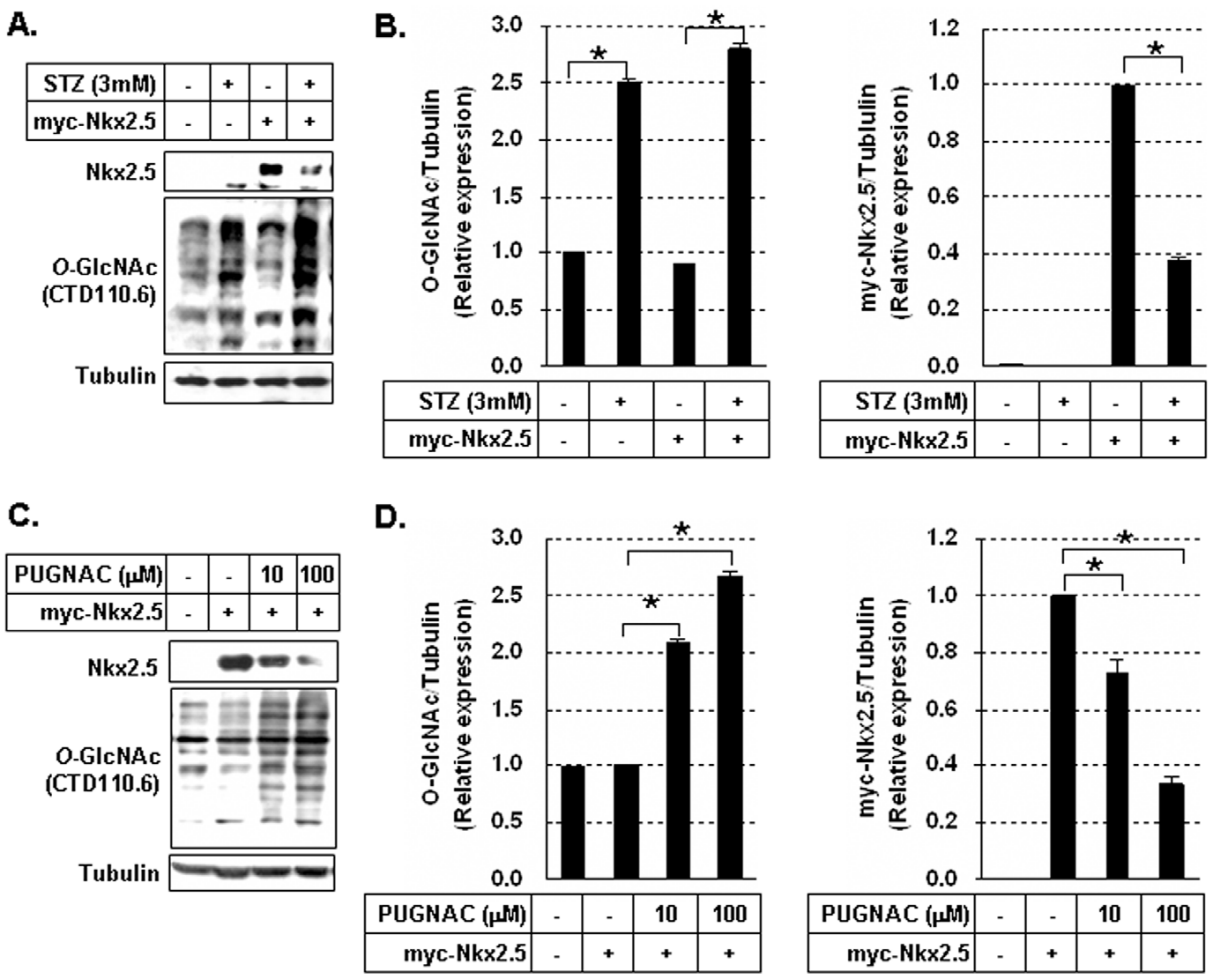
Reduction of myc-Nkx2.5 protein in cells treated with *O*-GlcNAcase inhibotirs. (A) Immunoblotting of myc-Nkx2.5 and *O*-GlycNAcylated proteins in myc-Nkx2.5-transfected HEK293 cells in the presence or absence of 3 mM streptozotocine (STZ). (B) Graphs showed relative *O*-GlcNAc (left) and myc-Nkx2.5 (right) levels normalized to the tubulin levels in STZ-treated and untreated cells. *O*-GlcNAcylation levels were compared in untreated cells *vs* STZ-treated cells and untreated myc-Nkx2.5 cells *vs* STZ-treated myc-Nkx2.5 cells. The myc-Nkx2.5 protein levels were compared in untreated myc-Nkx2.5 cells *vs* STZ-treated myc-Nkx2.5 cells. All values are presented as the mean±standard error of three independent experiments. *p<0.05. (C) Immunoblotting analysis of myc-Nkx2.5 and *O*-GlycNAcylated proteins in myc-Nkx2.5-transduced HEK293 cells in the presence or absence of *O*-(2-acetamido-2-deoxy-D-glucopyroanosylidene)-amino-*N*-phenylcarbamate (10 or 100 µM PUGNAC). (D) Graphs showed relative *O*-GlcNAc (left) and myc-Nkx2.5 (right) levels normalized to the tubulin levels in PUGNAC-treated and untreated cells. The treatment with STZ and PUGNAC significantly increased the *O*-GlcNAcylation of intracellular proteins, but decreased the myc-Nkx2.5 protein significantly. *O*-GlcNAcylation levels were compared in untreated myc-Nkx2.5 cells *vs* PUGNAC-treated myc-Nkx2.5 cells. The myc-Nkx2.5 protein levels were compared in untreated myc-Nkx2.5 cells *vs* PUGNAC treated myc-Nkx2.5 cells. All values are presented as the mean±standard error of three independent experiments. *p<0.05.

We investigated the effect of the *O*-GlcNAcase inhibitors, STZ and PUGNAC, on myc-Nkx2.5 protein levels in HEK293 cells. Immunoblotting analysis revealed that treatment with STZ (3 mM) for 24 hours remarkably increased the level of general protein *O*-GlcNAcylation, but significantly decreased the expression level of myc-Nkx2.5 in cells (Fig. 2A and B, *p*<0.05). We assessed the expression level of myc-Nkx2.5 in the presence of STZ by immunofluorescence. Myc-Nkx2.5 was detected in the nuclei of cells and at reduced levels in STZ-treated cells (data not shown). The other *O*-GlcNAcase inhibitor, PUGNAc increased the *O*-GlcNAcylation dose-dependently but also significantly decreased the expression of myc-Nkx2.5 (Fig. 2C and D, *p*<0.05).

### Nkx2.5 Interacted with OGT Proteins and was Modified by O-GlcNAc

**Figure 3:**
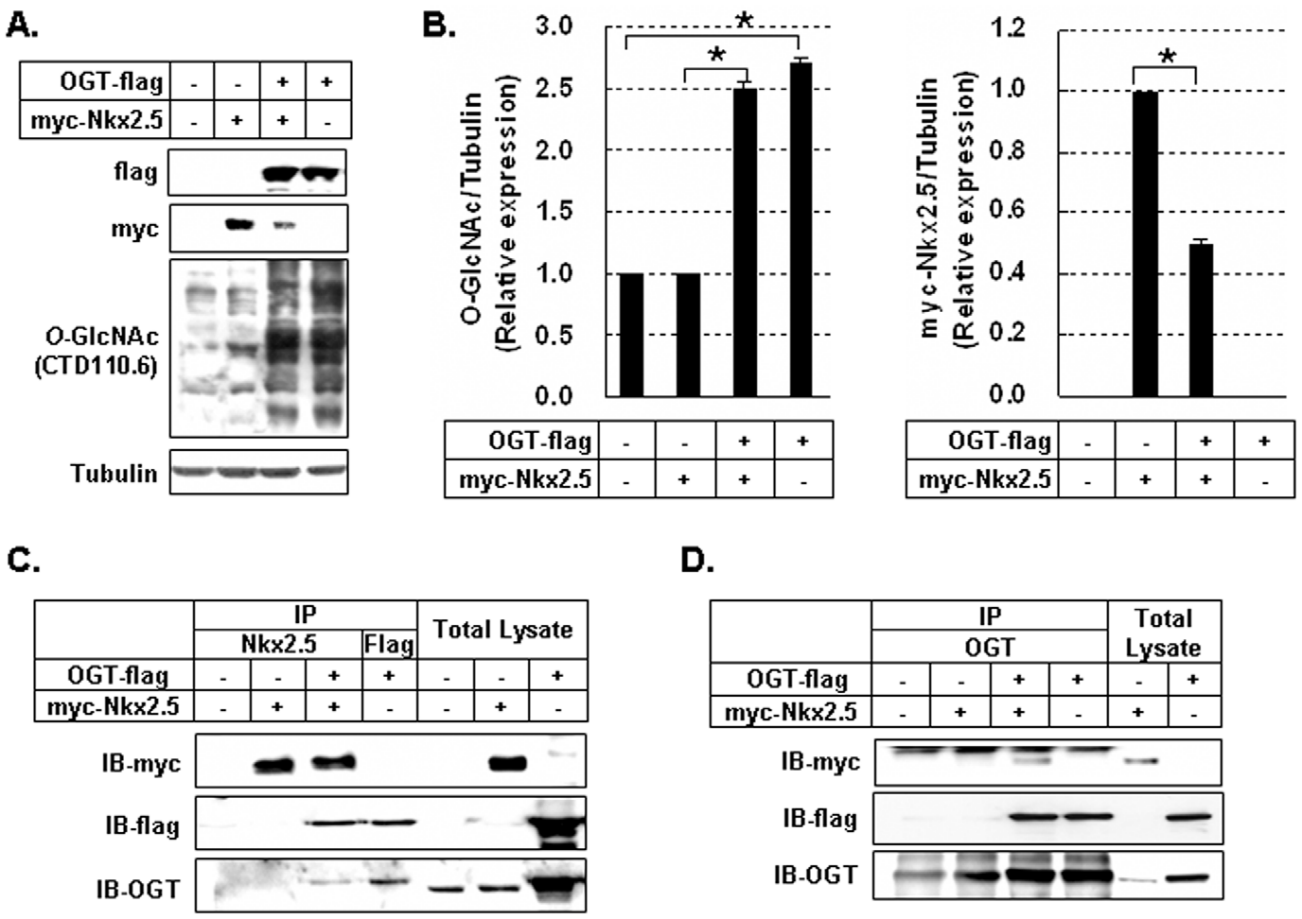
Interaction of Nkx2.5 and *O-*GlcNAc transferase (OGT). (A) Immunoblotting analysis of myc-Nkx2.5 and *O*-GlycNAcylated proteins in myc-Nkx2.5 and OGT-flag-co-transfected HEK293 cells. (B) Graphs showed relative *O*-GlcNAc (left) and myc-Nkx2.5 (right) levels normalized to the tubulin levels in co-transfected cells and single transfected cells. *O*-GlcNAcylation levels were compared in no-transfected cells *vs* OGT-flag cells and myc-Nkx2.5 cells *vs* OGT-flag/myc-Nkx2.5 cells. Myc-Nkx2.5 protein levels were compared in myc-Nkx2.5 cells *vs* OGT-flag/myc-Nkx2.5 cells. All values are presented as the mean±standard error of three independent experiments. *p<0.05. (C) After immunoprecipitation with antibodies specific for Nkx2.5 and flag, immunoblotting with antibodies for myc, flag, and OGT was performed. (D) After immunoprecipitation with antibodies specific for OGT, immunoblotting with antibodies for myc, flag, and OGT was performed. The interaction between myc-Nkx2.5 and OGT-flag was observed.

To determine if excessive *O*-GlcNAcylation induced by OGT overexpression could reduce Nkx2.5 expression, OGT-flag and myc-Nkx2.5 genes was co-transfected into HEK293 cells and immunoblotting was performed. Myc-Nkx2.5 levels were significantly lower in HEK293 cells co-transfected with OGT-flag (Fig. 3A and B, *p*<0.05). The interaction of myc-Nkx2.5 protein and OGT-flag protein was assessed by co-immunoprecipitation. As shown in figure 3C and D, co-immunoprecipitation analysis demonstrated that myc-Nkx2.5 protein interacted with OGT-flag protein.

**Figure 4:**
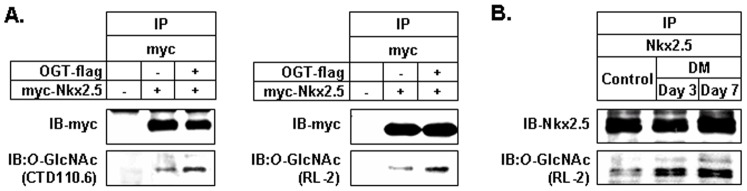
Modification of Nkx2.5 by *O*-GlcNAc. (A) The lysates of myc-Nkx2.5 and OGT-flag-co-transfected HEK293 cells were subjected to immunoprecipitation with anti-myc antibody and immunoblottings were performed with antibodies against *O*-GlcNAc (CTD110.6, RL-2) and myc. The modification of myc-Nkx2.5 with O-GlcNAc was detected. (B) Diabetic mice (DM) maintained elevated blood glucose level (>430 mg/dL) at 3 and 7 days after injection with streptozotocine (180 mg/kg body weight). The heart homogenates of control and diabetic mice were immunoprecipitated with anti-Nkx2.5 antibody, followed by immunoblottings with RL-2 antibodies against *O*-GlcNAc and anti-Nkx2.5 antibody. Nkx2.5 proteins of heart tissues were modified with *O*-GlcNAc and this modification increased in diabetic mice compared with control mice.

We further investigated whether the interaction of Nkx2.5 protein with OGT protein can directly induce *O*-GlcNAcylation of myc-Nkx2.5. After immunoprecipitation of myc-Nkx2.5 proteins with antibody for myc, immunoblotting for *O*-GlcNAc was performed with CTD110.6 and RL-2 antibodies. We found that myc-Nkx2.5 protein was gylcocsylated with *O*-GlcNAc (Fig. 4A). Next, we determined if Nkx2.5 protein of heart tissues is modified with *O*-GlcNAc. Heart homogenates were subjected to immunoprecipitation with anti-Nkx2.5 antibody, followed by immunoblotting with anti-*O*-GlcNAc antibodies, RL-2. STZ-induced diabetic mice with glucose levels of greater than 400 mg/dL at 3 and 7 days post-injection were used. Nkx2.5 proteins were modified with *O*-GlcNAc and this modification in Nkx2.5 proteins was increased in diabetic heart compared with control heart (Fig. 4B).

## Discussion

Elevated *O*-GlcNAcylation has been implicated in the development of diabetic cardiomyopathy [2], [9], [11], [20]. While the contributing mechanism is not clear, *O*-GlcNAcylation modification of cardiac myofilament proteins (MLC2 and TnI) may modulate myocardial contractile function, and chronic exposure to elevated *O*-GlcNAcylation may impair cardiac function in diabetic patients [21]. Moreover, elevated *O*-GlcNAcylation induces collagen expression, which results in diabetic cardiomyopathy accompanying by myocardial fibrosis [22]. Here we show that the induction of Nkx2.5 downregulation in response to *O*-GlcNAcylation conditions may be an additional diabetic cardiomyopathy mechanism and possibly interfere with cardiomyocyte survival pathways.

Nkx2.5 is essential for embryonic cardiogenesis, abundantly expressed in the adult heart, and required for homeostasis and survival of cardiac myocytes in the adult heart [12]–[14], [16], [23]. The transgenic mice that overexpress a dominant negative mutant of Nkx2–5 showed degeneration of cardiac myocytes and impairment of cardiac function in adult heart, and Nkx2.5 was critical for maintaining a highly differentiated cardiac phenotype and for protecting the heart from stresses [23].

Relatively little is known about the post-translational regulation of Nkx2.5 protein. On serine 163 of Nkx2.5 protein is phosphorylated by casein kinase II, which is involved in nuclear localization and transcriptional activity of Nkx2.5 protein [18]. We previously demonstrated that the differentiation of Nkx2.5-lineage cardiomyocyte precursor cells from ES cells was suppressed in excessive *O*-GlcNAcylation conditions [17].

The modification of Nkx2.5 proteins with *O*-GlcNAc as well as the role *O*-GlcNAc in the function of Nkx2.5 proteins was not known. To understand the role of *O*-GlcNAc in post-translational regulation of Nkx2.5, it would be important to extend such an approach to cultured adult cardiac myocytes. However, the limitations involved with adult cardiac myocytes are the absence of mitosis and possibility of these cells to proliferate and therefore procurement of a large quantity of cells is almost impossible to study compared the drug effect on *O*-GlcNAcylation in cardiomyocytes isolated from the same individual. In the present study, we showed that Nkx2.5 proteins were modified with *O*-GlcNAc and the excessive *O*-GlcNAcylation conditions result in the downregulation of Nkx2.5 protein. This effect was detectable in transfected HEK293 cells and in the heart tissues of STZ-induced diabetic mice with glucose levels of greater than 400 mg/dL.

Computer-assisted program, YinOYang 1.2 can predict potential sites in a protein on which phosphorylation and *O*- GlcNAcylation compete with one another for the same site. According to results obtained from YinOYang 1.2, there were 13 sites in Nxk2.5 predicted as potential Yin Yang sites (having same serine/threonine residues for both phosphorylation and *O*-GlcNAcylation). We suggest that excessive O-GlcNAcyation in Nkx2.5 protein may alter its stability via the “Ying-Yang hypothesis” whereby O-GlcNAcylation may be competitive or reciprocal to phosphorylation, which can lead to a decrease in Nkx2.5 protein level.

Critical goals of future studies are to define the regulatory pathways that control the downregulation of Nkx2.5 in response to excessive *O*-GlcNAcylation and the pathways downstream of Nkx2.5 that are required for cardiomyocyte development and function.

## Materials and Methods

### Reagents and Antibodies

STZ and PUGNAc for inhibiting *O*-GlcNAcase were purchased from Sigma (St Louis, MO) and Toronto Research Chemicals (North York, Ontario, Canada). The plasmid expressing recombinant mouse Nkx2.5 gene (myc-Nkx2.5) was provided by Dr. Hidaka (National Cardiovascular Research Institute, Osaka, Japan). The plasmid expressing recombinant human OGT (OGT-flag) was provided by Dr. Cho (Yeonsei University, Seoul, Korea). Antibodies against Nkx2.5, myc, and tubulin were purchased from Santa Cruz Biotechnology (Santa Cruz, CA). The antibodies CTD110.6 and RL-2 for *O-*GlcNAc detection was purchased from Covance (Princeton, NJ) and Thermoscientific (Billerica, MA), respectively. Antibodies against OGT and FLAG were purchased from Sigma.

### Transfection with Myc-Nkx2.5 and OGT-flag Plasmid

Human embryonic kidney (HEK293) cells were obtained from the American Type Culture Collection (ATCC, Rockville, MD). HEK293 cells were cultured in Dulbecco’s modified Eagle’s medium (DMEM; Life Technologies, Invitrogen, Cergy Pontoise, France) supplemented with 10% fetal bovine serum (FBS) (v/v), 2 mM L-glutamine, 5 IU/ml penicillin, and 50 µg/ml streptomycin at 37°C in a humidified atmosphere enriched with 5% CO_2_. HEK293 cells were treated with one of the following OGA inhibitors STZ (1–3 mM) or PUGNAc (10–100 µM). HEK293 cells were transiently transfected with myc-Nkx2.5 or the OGT-flag plasmids using Lipofectamine and Plus Reagent (Invitrogen). Myc-Nkx2.5 and OGT-flag expression was confirmed by immunoprecipitation and immunoblotting.

### Immunoblotting

HEK293 cells were washed with cold phosphate-buffered saline (PBS), and then lysed in lysis buffer containing a protease inhibitor mixture (0.1 mM phenylmethylsulfonyl fluoride, 5 µg/ml aprotinin, 5 µg/ml pepstatin A, and 1 µg/ml chymostatin). Proteins were separated by SDS-PAGE and transferred to nitrocellulose membranes. The membrane was blocked with 5% nonfat dry milk in Tris-buffered saline and then incubated with primary antibodies. Blots were developed using peroxidase-conjugated secondary antibodies and visualized with enhanced chemiluminescence reagent (Amersham Biosciences, Buckinghamshire, UK) according to the manufacturer's recommendations.

### Immunoprecipitation

For immunoprecipitation experiments, cell extracts and heart homogenates were centrifuged at 20,000 *g* for 30 minutes at 4°C. Aliquots of 500 µg proteins extracted cells and heart tissues were incubated with mouse monoclonal anti-Nkx2.5, anti-OGT, anti-flag, anti-myc or anti-*O*-GlcNAc antibodies at 4°C overnight. Antibody-bound proteins were precipitated with 30 µl of A/G protein-coupled sepharose beads for 1−4 hours at 4°C. Beads were gently centrifuged for 1 minute and washed with lysis buffer. Finally, 25−50 µl of loading buffer was added and the beads boiled at 95−100°C for 5 minutes to denature the protein and separate it from the protein-A/G beads. Immunoblotting of the recovered protein was performed.

### Immunofluorescence Staining

HEK293 cells were grown on 8-well chamber slides and washed twice with cold PBS. Cells were fixed in 3% of paraformaldehyde in cold PBS for 15 minutes, washed with PBS, and permeabilized with 0.1% Triton X-100 for 5 minutes. Nonspecific sites were blocked with goat serum. Fixed cells were then incubated for 60 minutes with anti-myc antibody and myc-Nkx2.5. Labeling was visualized using FITC-conjugated secondary antibodies. Nuclei were specifically stained with 4′,6′-diamidino-2-phenylindole (DAPI). Images of stained cells were acquired with a fluorescence microscopy (Leica, Wetzlar, Germany), equipped with a CCD camera (Leica) using a green filter (excitation 488 nm, emission 520 nm).

### Diabetic Mouse Model

C57BL/6 mice were supplied from the Center for Animal Resource and Development, Seoul National University. The mice used in this study, all between 4 and 20 weeks of age, were maintained in individually ventilated cages (Thoren caging systems, Hazleton, PA) at 24±2°C and 50±5% humidity with a 12 h/12 h (light/dark) cycle. Mice were given ad libitum access to irradiated mouse feed (Purina Korea, Seoul, Korea) and reverse osmosis water containing 2 ppm chloride. For diabetic mice, seven-week-old male C57BL/6 mice were intraperitoneally injected with 180 mg of STZ per kg of body weight. The blood glucose levels of mice were measured daily, and mice with blood glucose levels of 300−500 mg/dL were considered to be diabetic. Collected heart tissues were homogenized in lysis buffer for immunoblotting. Non-diabetic control heart tissue was obtained age-matched male C57BL/6 mice.

### Ethics Statement

Additionally, all animal experiments were conducted in the Center for Animal Resource and Development. This research was approved by the Institutional Animal Care and Use Committee (IACUC) of Seoul National University (SNU-080828-2).

### Statistical Analysis

Data are presented as the means±S.E.M. for at least three independent experiments and statistically evaluated using ANOVA followed by the t-test. *P*-values less than 0.05 were considered statistically significant.
